# Temporal Trends in the Epidemiology of Eating Disorders Between 2000 and 2022: A Danish Register Study of Their Incidence and Comorbidities

**DOI:** 10.1002/erv.70061

**Published:** 2025-12-16

**Authors:** Nadia Micali, Helena L. Davies, Heidi Jeannet Graff, Laust H. Mortensen, Emilie R. Hegelund

**Affiliations:** ^1^ Center for Eating and Feeding Disorders Research Mental Health Center Ballerup Copenhagen University Hospital—Mental Health Services CPH Copenhagen Denmark; ^2^ Institute of Biological Psychiatry Mental Health Center Sct. Hans Mental Health Services Copenhagen Roskilde Denmark; ^3^ Great Ormond Street Institute of Child Health UCL London UK; ^4^ ROCKWOOL Foundation Copenhagen Denmark; ^5^ Department of Public Health University of Copenhagen Copenhagen Denmark; ^6^ Data Science Lab Statistics Denmark Copenhagen Denmark

**Keywords:** comorbidity, COVID‐19, eating disorders, epidemiology, incidence, prevalence

## Abstract

**Background:**

Eating disorders are debilitating illnesses that often co‐occur with other psychiatric disorders and somatic diseases. Evidence indicates that the incidence of eating disorders has been increasing. We first examine the landscape of EDs over time, including the COVID‐19 period, via assessing the incidence of anorexia nervosa (AN), bulimia nervosa (BN), and other eating disorders (OED) in Denmark. We additionally map the impact of eating disorders by assessing their prevalence and comorbidities.

**Methods:**

Diagnosed eating disorder cases were identified from the Danish National Patient Register from 1995 to 2022. We calculated age‐ and sex‐specific incidence rates for each year. We additionally calculated prevalence for the years 2000, 2010, and 2022 and identified comorbidities via primary or secondary ICD‐10 diagnoses from inpatient and outpatient hospital contacts and prescription medication data from the Danish National Prescription Registry. Associations between eating disorders and ICD‐10 diseases and prescription medication were investigated with logistic regression models.

**Results:**

The incidence of eating disorders increased over the study time in younger age groups for both sexes, particularly for AN and OED, whilst BN diagnoses showed a declining incidence rate (IR) from 2017 onwards. Evidence for increased incidence rates during and following the COVID‐19 pandemic was strongest for AN and OED in females aged 10–14 (respectively a 35.5% increase in IR for AN and 57.1% for OED between 2019 and 2021). All eating disorders showed high levels of comorbidities with both psychiatric and somatic illnesses. For example, BN (OR = 23.37, 95% CI: 17.52–31.16) and OED (OR = 19.09, 95% CI: 14.89–24.48) were associated with subsequent abuse of non‐dependence‐producing substances, and AN was associated with diseases of the circulatory system (OR = 1.88, 95% CI: 1.77–2.01), with diagnoses occurring on average almost 1 year after AN.

**Discussion:**

The incidence of AN and OED has increased in the last 22 years in Denmark. Increased incidence in younger age groups after 2020 is likely due to psychosocial challenges and heightened vulnerability to mental health difficulties during the COVID‐19 pandemic. Low prevalence of EDs in males may demonstrate poor identification and underdiagnosis. EDs have high public health impact given their increasing prevalence and breadth of identified somatic and psychiatric comorbidities.

## Highlights

- New diagnoses of EDs have increased in Denmark over the last 22 years. This is particularly true for AN and OED in young females, whilst there is evidence of a decrease in incidence of BN.
- This is the first comprehensive study to confirm an increase in new OED diagnoses and an increase in AN cases during the COVID‐19 pandemic at the population level. These increases are specific to girls/young women below the age of 20 years.
- Apart from their prevalence, the impact of EDs at a healthcare level is further confirmed by their high level of comorbidity with many physical and mental health illnesses, which warrants a detailed investigation of timing of occurrence.

## 1 Introduction

Eating disorders (EDs) are severe mental health conditions linked to significant psychiatric and medical complications, as well as elevated mortality rates (Arcelus et al. 2011; Momen et al. 2022; H. Steinhausen et al. 2021). Though relatively rare (Keski‐Rahkonen and Mustelin, 2016; Smink et al. 2012; Swanson et al. 2011), the prevalence and incidence of EDs have risen, particularly among young females and for anorexia nervosa (AN) diagnoses (Galmiche et al. 2019; Hoek 2006). In Denmark, recent studies have reported a cumulative ED incidence of 1.85% in a cohort of people born between 1981 and 2009 (Larsen et al. 2024). Another Danish health register study (2004–2021) found an overall ED incidence of 2.64 and an AN incidence of 0.78 per 10,000 person‐years, with both increasing over time (Momen et al. 2024). While incidence rates for AN (Momen et al. 2024; H.‐C. Steinhausen and Jensen 2015) and bulimia nervosa (H.‐C. Steinhausen and Jensen 2015) (BN) have grown since 1995, other EDs, including EDNOS (eating disorder not otherwise specified), contribute significantly to the population‐level burden (Santomauro et al. 2021). Notably, the incidence of EDNOS has increased in Danish adolescents (Zerwas et al. 2015), with incidence rates almost as high as AN in females, and higher than AN for males (Larsen et al. 2024).

Outside Denmark, ED incidence rates have risen in the UK during the first decade of this century (Micali et al. 2013). UK studies on temporal trends using primary care registers highlight significant increases among girls, particularly those aged 13–16 years, in the first 2 decades of 2000 (Cybulski et al. 2021; Trafford et al. 2023), making EDs the third most common mental health disorder in adolescent girls (Trafford et al. 2023).

Monitoring trends in ED prevalence and incidence measures disorder burden and helps plan services to improve public health (GBD 2019 Diseases and Injuries Collaborators et al. 2020). The negative effects of the COVID‐19 pandemic on mental health have been widely discussed (World Health Organization 2022), with temporary increases in AN and BN cases observed in 13‐24‐year‐old Danes (Sonne et al. 2024), however, this study did not stratify by sex or assess EDs other than AN or BN. Similar results have been found in Norway, with 46% higher than expected incidence rates of new‐onset EDs in 2021 for females (Reas et al. 2025), and in Germany via health insurance data, with 40% higher hospital admission rates for AN in girls in 2023 than before the COVID‐19 pandemic (Herpertz‐Dahlmann et al. 2024). In contrast, however, data from the Danish National Birth Cohort indicated a slight decrease in weight and shape concern, binge eating, self‐induced vomiting, laxative use, or threshold EDs during or after COVID‐19 lockdowns in Denmark among women, with no change in men (Danielsen et al. 2023). This study focused mainly on young adults living with parents (mean age higher than the age group affected by an increase in ED diagnoses in register‐based studies), and used self‐reported survey data which is more sensitive to drop‐out due to mental health problems than register data.

We aimed to investigate sex‐ and age‐stratified incidence rates of AN, BN, and other EDs (OED, which broadly captures DSM‐IV EDNOS), and specifically to study potential increases in incidence during and after the COVID‐19 pandemic. We aimed to expand upon prior research by leveraging Danish nationwide register data through 2022. We hypothesised: (1) an increasing trend in ED incidence during the study period, particularly among younger females, and (2) a peak in ED incidence in 2021–2022, following the initial COVID‐19 outbreak.

To assess the impact of EDs, we additionally calculated the prevalence of all three EDs in the years 2000, 2010, and 2022. Further, given evidence from Danish and Swedish register‐based studies that AN frequently co‐occurs with psychiatric (H. Steinhausen et al. 2021; Cederlöf et al. 2015; Clausen et al. 2023; Kask et al. 2016, 2017; Koch et al. 2015; Meier et al. 2015; Papadopoulos et al. 2009; Zhang et al. 2021) and somatic illnesses (Clausen et al. 2023; Papadopoulos et al. 2009; Ji et al. 2016) and the limited research on BN and OED (Hedman et al. 2019; Mellentin et al. 2022; Shen et al. 2023; Skøt et al. 2022; Yao et al. 2016), we leveraged Danish registers to better quantify the burden of EDs by exploring comorbidities across all diagnosed EDs. Comorbidities contribute to the high mortality, healthcare burden, and underdiagnosis of EDs, highlighting the need for early detection, integrated care, and targeted prevention strategies. Identifying the overlap with other conditions can also enhance understanding of shared aetiology, help refine diagnostic tools, and can inform models of risk, particularly given our focus on their temporal relationship. We hypothesised that individuals with EDs would show elevated psychiatric (e.g., OCD, depression) and somatic (e.g., autoimmune, gastrointestinal disorders) comorbidities and that their prevalence would increase over time.

## 2 Methods

### 2.1 Study Population

This nationwide register‐based cohort study included all Danish citizens aged 10 or older, identified via the Danish Civil Registration System (CRS) (Pedersen 2011), who lived in Denmark at any point between 1 January 1995 and 31 December 2022. Using the unique central personal registry (CPR) number (M. Schmidt et al. 2014), we linked to the Danish National Patient Register (DNPR) (Lynge et al. 2011), which has recorded inpatient admissions since 1977 and outpatient admissions since 1995. Since 1994, the DNPR has used ICD‐10 diagnostic codes (M. Schmidt et al. 2015).

### 2.2 Case Definition of Eating Disorders

Incident cases were identified based on the date of diagnosis and defined as having a primary (i.e., the reason for the patient's hospital visit or the most resource‐intensive condition) or secondary (i.e., a co‐existing condition at the time of hospital visit) ICD‐10 diagnosis of an ED, including AN (F50.0, F50.1), BN (F50.2, F50.3), and OED (F50.8, i.e., other EDs [including binge‐eating disorder and other specified EDs] and F50.9, i.e., ED unspecified, including feeding or ED unspecified) in DNPR (Lynge et al. 2011). These codes are assigned after in‐ or outpatient hospital contact in Denmark. Patients with multiple ED diagnoses were analyzed under each relevant ED category. For the overall ED category, we included only the first occurrence in each of the categories (AN, BN and OED). Subsequent identical diagnoses are not counted. Therefore, the number of overall ED cases is slightly lower than the sum of AN, BN and OED cases. We included all data up until 31st December 2022.

### 2.3 Statistical Analyses

**Incidence:** Yearly incidence rates from 2000 to 2022 were calculated as new ED cases in a given calendar year divided by person‐years (PY) (per 10,000 PY) among individuals aged ≥ 10 years who had no prior ED diagnosis. Diagnoses below age 10 are likely to be a misdiagnosis or capture ARFID or paediatric feeding disorders, which are not currently recognised diagnoses in the Danish system and are not a focus of this study. Analyses were stratified by sex and 5‐year age groups for each ED type and overall. The 5‐year age group balanced granularity with clinical relevance, reflecting meaningful developmental stages in the onset and course of EDs. To assess the impact of COVID‐19, incidence rates were examined yearly for 2018–2022, categorising 2018–2019 as ‘pre‐pandemic’, 2020–2021 as ‘during pandemic’, and 2022 as ‘post‐pandemic’.

To test if the sex‐ratio incidence changed over time, we tested the interaction between sex and calendar year in a generalised additive Poisson regression model where calendar year was modelled as a smoothing spline with the effective degrees of freedom selected using generalised cross‐validation. To test the changes in the age‐specific incidence before, during, and after COVID‐19, we restricted the data to age‐groups 10–24 as they were followed throughout this period. We then tested a model where we assumed no change in rates against a generalised additive Poisson regression model where calendar year was modelled as a smoothing spline with the effective degrees of freedom selected using generalised cross‐validation. We ran the models by sex. This tests if there is change in rates across the COVID‐19 pandemic but does not test trends in age‐specific changes. We note that since the numbers reported are full‐population statistics, the tests and confidence intervals cannot be interpreted as sampling error.

**Prevalence:** Age and sex‐specific estimates of prevalence were calculated as the number of cases registered with an ED diagnosis in connection with a hospital contact (AN, BN, or OED) in the years 2000, 2010, and 2022 divided by the number of individuals alive and living in Denmark aged 10 years or above on 31st December 2000, 2010, and 2022, respectively.

As information on EDs from outpatient hospital facilities was not available until 1995, we only have complete follow‐up data on individuals aged ≥ 10 years for those born since 1985. Since 1 January 2000 is the first date by which all individuals in the 1985 birth cohort have turned at least 14 years, which is the upper limit for our youngest 5‐year age group (i.e., 10–14 years), we have chosen only to present prevalence and incidence rates between 2000 and 2022.

**Somatic and psychiatric comorbidities:** Somatic and psychiatric comorbidities were extracted from the DNPR as primary or secondary ICD‐10 diagnoses from inpatient and outpatient contacts between 1995 and 2022. To cover comorbidities not captured in the DNPR, we included prescription data from the Danish National Prescription Registry (Kildemoes et al. 2011), which includes information on all redeemed prescriptions at community pharmacies with product type and Anatomical Therapeutic Chemical classification (ATC) code. The ATC has five levels, ordered hierarchically. The first level classifies drugs according to the main anatomical or pharmacological group, for example, ‘Alimentary tract and metabolism’. The second level further classifies the drugs according to their therapeutic or pharmacological action, for example, ‘beta‐blockers’. Redeemed prescriptions were categorised according to the ATC system and the three‐digit ATC codes (level 2).

We used logistic regression models with AN, BN, or OED as exposures and other ICD‐10 diagnoses or ATC codes as outcomes. We examined both broader chapter‐level categories (e.g., Chapter V Mental and behavioural disorders) and specific sub‐categories (e.g., F00 Dementia in Alzheimer disease) in the ICD‐10 and ATC systems. We included all ICD‐10 chapters bar XV (Pregnancy, childbirth and the puerperium) and XVI (Certain conditions originating in the perinatal period) because they are related to any pregnancy the index individual might have and these analyses might be biased by a lower likelihood to become pregnant in individuals with EDs We included all ATC categories with no exclusions. The reference group included individuals without an ED diagnosis between 1995 and 2022. We conducted separate analyses on two medications: anxiolytics (N05B) and antidepressants (N06A) to capture milder mental health disorders (other psychotropics are prescribed in secondary or tertiary care).

We additionally report the median age at first diagnosis of the comorbidity (for all individuals, regardless of ED diagnosis to get an idea of the typical timing of the onset of the disease in question) and the median number of years elapsed between each ED diagnosis and other illnesses. The latter indicates if half or more of the individuals with EDs and a given comorbidity were diagnosed with the comorbidity *before* the ED diagnosis (negative value) or *after* the ED diagnosis (positive value). We report false discovery rate *q*‐values to take account of multiple testing (< 0.05 considered statistically significant). We adjusted all regression models for year of birth and sex unless there were fewer than 100 male or female cases, in which case we restricted our analyses to the sex with ≥ 100 cases and did not adjust for sex. R studio 4.2.3 was used for all statistical analyses.

## 3 Results

### 3.1 Incidence of EDs

**FIGURE 1:**
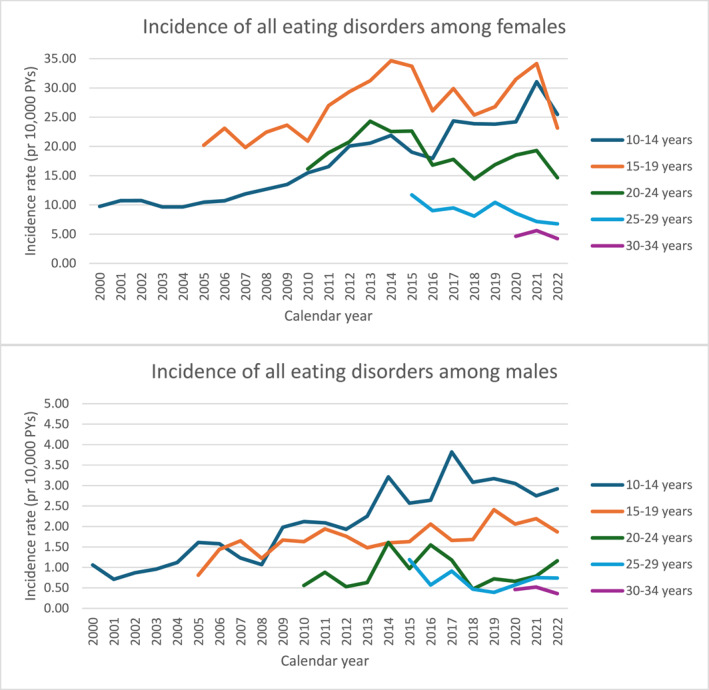
Female (top) and male (bottom) incidence rate per 10,000 person‐years (PY) of all eating disorders (anorexia nervosa, bulimia nervosa, and other eating disorders) in the years 2000–2022, stratified by age groups (10–14 years; 15–19 years; 20–24 years; 25–29 years; 30–34 years). Incidence is calculated via the number of new events in the given calendar year divided by the number of PY per 10,000 amongst the population at risk, individuals aged ≥ 10 years on 1 January in the given year who had not previously been diagnosed with an eating disorder.

The incidence rates (IRs) of all EDs were highest for females and increased over time, particularly in younger age groups (Table 1; Figure 1; see Supporting Information S3: Appendix for further details). For example, the IRs of all EDs for females aged 10–14 years steadily increased from 9.74 (2000) to 15.48 (2010) to 25.47 per 10,000 PY (2022); and for girls aged 15–19 from 20.91 (2010) to 33.72 (2015) to 34.16 (2021), before dropping to 23.14 per 10,000 PY (2022). Males also had an increasing incidence of all EDs in the younger age groups, particularly 10–14 years of age, with IR increases from 1.06 to 2.92 per 10,000 PY from 2000 to 2022, an almost three‐fold increase.

**TABLE 1:** Sex‐and‐age stratified incidence rates of anorexia nervosa (AN), bulimia nervosa (BN), other eating disorders (OED), and all combined (AN + BN + OED) in the years 2000–2022.

Calendar year	2000	2001	2002	2003	2004	2005	2006	2007	2008	2009	2010	2011	2012	2013	2014	2015	2016	2017	2018	2019	2020	2021	2022
Females																							
Anorexia nervosa																							
10–14 years	6.16	6.52	7.38	5.97	6.31	6.95	6.77	7.73	7.64	9.37	10.46	10.50	11.38	14.19	14.37	12.67	11.83	16.06	15.13	16.47	16.85	22.33	19.71
15–19 years						8.62	10.29	11.19	11.83	11.90	10.87	13.12	14.12	17.01	19.05	18.14	13.01	16.70	13.78	14.52	16.17	21.05	12.96
20–24 years											6.09	7.57	8.19	10.43	9.75	9.72	7.12	7.97	6.01	8.38	8.08	7.85	7.30
25–29 years																3.39	3.12	3.92	3.62	2.96	3.57	2.28	2.93
30–34 years																					1.66	1.76	1.39
Bulimia nervosa																							
10–14 years	1.54	1.36	1.05	1.40	0.99	1.15	1.13	1.12	1.30	0.77	0.84	0.85	1.72	1.49	1.37	0.94	0.75	0.94	0.87	0.99	0.87	1.42	0.82
15–19 years						7.95	7.83	5.91	7.38	7.75	6.70	7.56	8.46	8.49	9.40	8.97	6.88	8.19	6.13	5.14	6.55	5.48	4.89
20–24 years											6.60	9.66	10.14	12.02	11.57	12.59	7.53	8.68	7.17	6.96	6.58	7.58	4.81
25–29 years																7.01	3.89	5.26	4.26	3.79	3.28	3.07	2.65
30–34 years																					1.82	2.07	1.23
Other eating disorders																							
10–14 years	3.43	4.41	4.02	4.06	4.02	4.23	4.40	4.49	5.98	5.55	6.28	8.29	11.25	9.89	11.61	10.09	8.68	13.49	12.20	12.44	14.68	19.54	12.77
15–19 years						9.25	10.27	8.68	9.40	10.61	10.11	13.46	14.40	15.17	18.28	16.62	14.98	15.73	12.04	17.76	22.63	24.56	16.60
20–24 years											8.36	10.20	9.95	10.06	10.54	10.10	9.03	8.08	6.13	13.50	16.26	15.97	12.70
25–29 years																6.45	4.91	5.12	3.69	8.59	7.61	8.13	6.33
30–34 years																					4.57	5.12	4.02
All eating disorders																							
10–14 years	9.74	10.73	10.74	9.66	9.66	10.46	10.70	11.88	12.68	13.49	15.48	16.55	20.07	20.56	21.89	19.02	17.95	24.37	23.88	23.81	24.20	31.06	25.47
15–19 years						20.20	23.11	19.82	22.44	23.64	20.91	26.97	29.35	31.23	34.65	33.72	26.08	29.89	25.37	26.80	31.47	34.16	23.14
20–24 years											16.14	18.92	20.78	24.31	22.54	22.63	16.80	17.79	14.43	16.89	18.52	19.29	14.65
25–29 years																11.71	9.01	9.48	8.08	10.44	8.57	7.16	6.76
30–34 years																					4.64	5.61	4.24
Males																							
Anorexia nervosa																							
10–14 years	0.47	0.39	0.56	0.54	0.82	0.92	0.73	0.67	0.67	1.02	1.15	1.33	1.00	1.60	1.55	1.25	1.20	1.91	1.24	2.00	2.11	1.58	1.73
15–19 years						0.14	0.65	0.63	0.86	0.72	0.81	1.03	1.02	0.74	0.86	0.52	0.76	0.83	0.60	1.15	0.66	1.33	0.72
20–24 years											0.49	0.54	0.26	0.25	0.50	0.36	0.59	0.59	0.18	0.18	0.18	0.43	0.31
25–29 years																0.15	0.29	0.35	0.13	0.07	0.06	0.19	0.31
30–34 years																					0.15	0.07	0.00
Bulimia nervosa																							
10–14 years	0.07	0.00	0.00	0.00	0.06	0.06	0.00	0.06	0.00	0.00	0.06	0.00	0.00	0.06	0.12	0.00	0.00	0.06	0.12	0.00	0.00	0.00	0.00
15–19 years						0.20	0.26	0.25	0.24	0.18	0.17	0.17	0.34	0.23	0.17	0.23	0.18	0.36	0.06	0.06	0.30	0.00	0.12
20–24 years											0.07	0.14	0.00	0.13	0.31	0.06	0.36	0.12	0.24	0.24	0.18	0.12	0.37
25–29 years																0.30	0.14	0.21	0.13	0.00	0.25	0.06	0.06
30–34 years																					0.31	0.15	0.07
Other eating disorders																							
10–14 years	0.53	0.51	0.50	0.66	0.41	0.80	0.96	0.67	0.62	1.24	1.09	1.16	1.35	1.01	2.20	1.61	1.98	2.44	2.01	2.11	1.82	1.93	1.97
15–19 years						0.54	0.72	0.82	0.43	0.89	0.87	1.20	0.96	0.91	1.20	1.33	1.29	1.13	1.38	2.05	1.63	1.76	1.45
20–24 years											0.49	0.41	0.66	0.44	1.05	0.85	0.77	1.06	0.30	0.66	0.60	0.67	0.92
25–29 years																0.74	0.22	0.49	0.27	0.39	0.38	0.56	0.49
30–34 years																					0.39	0.37	0.36
All eating disorders																							
10–14 years	1.06	0.71	0.87	0.96	1.12	1.61	1.58	1.23	1.07	1.98	2.12	2.09	1.93	2.25	3.21	2.57	2.64	3.82	3.08	3.17	3.05	2.75	2.92
15–19 years						0.81	1.44	1.65	1.22	1.67	1.63	1.94	1.76	1.48	1.60	1.63	2.06	1.66	1.68	2.41	2.06	2.19	1.87
20–24 years											0.56	0.88	0.53	0.63	1.61	0.97	1.55	1.18	0.47	0.72	0.66	0.79	1.16
25–29 years																1.19	0.57	0.91	0.47	0.39	0.57	0.75	0.74
30–34 years																					0.46	0.52	0.36

*Note:* Incidence is calculated via the number of new events in the given calendar year divided by the number of PY per 10,000 amongst the population at risk, individuals aged ≥ 10 years on 1 January in the given year who had not previously been diagnosed with an eating disorder.

Whilst the highest IR was for 15–19‐year‐old females for OED in 2021 (24.56 per 10,000 PY) (Table 1; Supporting Information S1: Figure S1), AN most consistently had the highest IRs, especially among females and for ages between 10 and 14 since 2017 (Table 1; Supporting Information S1: Figure S2). Moreover, the incidence of AN increased dramatically between 2000 and 2022 (from 6.16 to 19.71 per 10,000 PY for 10–14‐year‐old females), a more than three‐fold increase in incidence. Males had the same increasing trend in incidence of AN, from 0.47 (2000) to 1.73 (2022) per 10,000 PY in 10–14‐year‐olds—a four‐fold increase ‐ and from 0.14 (2005) to 0.72 per 10,000 PY (2022) for 15–19‐year‐olds, a five‐fold increase (Table 1; Supporting Information S1: Figure S2).

The incidence of BN was highest among females aged 15–19 and 20–24 years but more recently shows a slight decrease (Table 1; Supporting Information S1: Figure S3). For example, the IR for 15–19‐year‐olds increased from 7.95 (2005) to 9.40 (2014), falling to 4.89 per 10,000 PY in 2022. Amongst males, similar to females, BN peaked in incidence in the age range 15–19 and 20–24 years, and decreased over time for the 15–19 years age group, albeit increasing in 2022 for the 20–24 years of age group (Table 1; Supporting Information S1: Figure S3).

We observed an increasing trend in incidence of OED in females, particularly for the 15–19 years age group (from 9.25 in 2005 to 16.60 per 10,000 PY in 2022), an almost two‐fold increase (Table 1; Supporting Information S1: Figure S1). Males also had a peak in incidence in the younger age groups (< 20 years; Table 1; Supporting Information S1: Figure S1). For example, the incidence of OED in 10–14‐year‐old males was 1.97 per 10,000 PY in 2022, an almost four‐fold increase from 2000.

In relation to sex ratios of ED incidence, the female:male ratio across all ages for AN was relatively stable over time ranging between 10.5:1 (in 2010) and 13.5:1 (in 2022). For BN, the female:male ratio increased steadily from 7.3:1 in 2000 to 14.7:1 in 2022. OED showed the lowest female:male ratio, and an increasing trend from 6.1:1 (2000) to 9.4:1 (2022). This is confirmed from our regression‐based test, which suggest change in the sex ratio over time for OED (*p* < 0.001) but not for AN (ns) and BN (ns).

### 3.2 Effect of COVID‐19 Pandemic on ED Incidence

The incidence of all EDs in females below the age of 25 years increased during the COVID‐19 period (See Table 1). Incidence peaked in 2021 with IRs of 31.06 and 34.16 per 10,000 PY for females aged 10–14 and 15–19, respectively. Although both groups showed increased IRs compared to pre‐pandemic, for the 15–19‐year‐old age group IRs dropped post‐pandemic (23.14 per 10,000 PY in 2022) to lower levels than pre‐pandemic; despite a drop from 2021 to 2022, IRs for 10–14‐year‐olds did not quite reach pre‐pandemic levels (23.81 per 10,000 PY in 2019, 31.06 per 10,000 PY in 2021 and 25.47 per 10,000 PY in 2022). IRs of all EDs for males were relatively stable for most age groups pre‐, during, and post‐pandemic. This interpretation was corroborated by tests for trend (*p* = ns for males; *p* < 0.001 for females).

For females in the younger age groups, the peaks in IRs were evident specifically for AN (e.g., 15.13 and 16.47 pre‐pandemic [2018 and 2019] to 22.33 per 10,000 PY during the pandemic [2021]) for those aged 10–14 years (Table 1). For males, the incidence of AN peaked for the younger age groups (< 20 years of age) pre‐pandemic (2019). Incidence in males remained fairly stable (despite some peaks and troughs) for all other age groups. This interpretation was corroborated by tests for trend (*p* = 0.05 for males; *p* < 0.001 for females).

IRs of BN followed a different pattern to AN, with slight increases in 2021 comparable to increases between 2012 and 2014 but remaining stable (or slightly decreasing for some age groups) for females from pre‐to post‐pandemic. The statistical tests for trend were not significant. IRs of BN in males show wide variation patterns probably due to low numbers, with an increase only visible for males aged 20–24 post‐pandemic (but to a level comparable to that of 2014 and 2016). This interpretation was corroborated by tests for trend which were non‐significant for males and females.

An increasing temporal trend in IRs was visible for OED for females in the two youngest age groups, with a peak in incidence in 2021 (19.54 per 10,000 PY in 10‐14‐year‐olds; 24.56 per 10,000 PY in 15–19‐year‐olds; Table 1), and a drop to pre‐pandemic levels for both 10–14 and 15–19‐year‐olds in 2022. IRs of OEDs were fairly stable from pre‐to post‐pandemic for males. This interpretation was corroborated by tests for trend (*p* = ns for males; *p* < 0.001 for females).

### 3.3 Impact of EDs

#### 3.3.1 Prevalence of EDs

**FIGURE 2:**
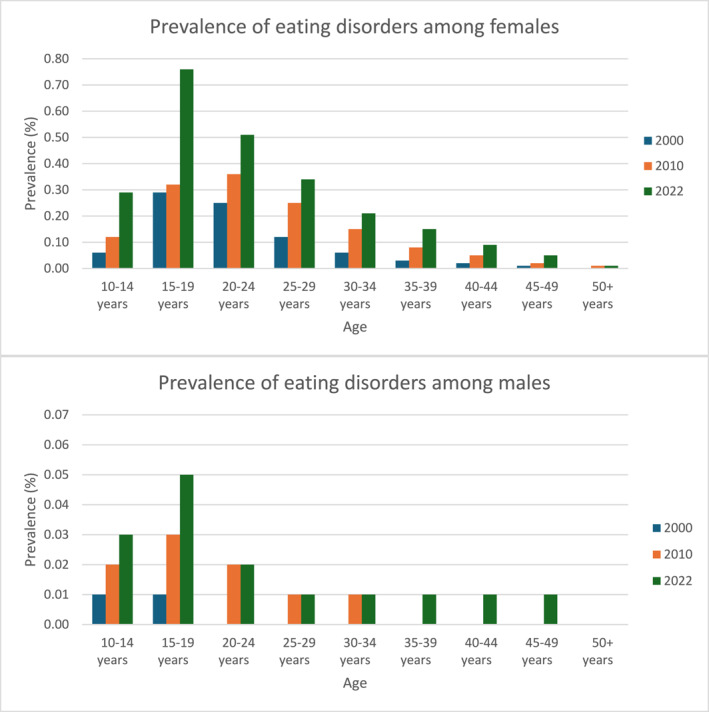
Female (top) and male (bottom) prevalence of all eating disorders (anorexia nervosa, bulimia nervosa, and other eating disorders) in the years 2000, 2010, 2022, stratified by age groups (10–14 years; 15–19 years; 20–24 years; 25–29 years; 30–34 years). Prevalence is calculated as the number of cases diagnosed with an eating disorder in the given year divided by the number of individuals alive and living in Denmark aged 10 years or above on 31st December in the given year.

Across the population under study, prevalences increased from 0.01% in 2000 for all three EDs to 0.05% for AN, 0.02% for BN, and 0.04% for OED in the year 2022 (Table 2). However, this trend was driven by females; for whom the prevalence of EDs increased from 0.06% in 2000 to 0.09% in 2010%, to 0.16% in 2022 whilst prevalence remained stable at 0% in 2000 to 0.01% in 2010 and 2022 in males (Table 2). Indeed, EDs were more prevalent in females than males across all years and age groups studied (Figure 2). We additionally report prevalence stratified by age and sex in Supporting Information S2: Tables S1–S3 and Supporting Information S1: Figures S4–S6.

**TABLE 2:** Point prevalence of eating disorders and age‐and‐sex stratified point prevalence of eating disorders (AN + BN + OED) among the Danish population aged ≥ 10 years on 31 December 2000, 2010, and 2022.

	2000	2010	2022
	(*N* = 4,660,255)	(*N* = 4,890,737)	(*N* = 5,305,742)
Total			
Anorexia nervosa	0.01	0.03	0.05
Bulimia nervosa	0.01	0.01	0.02
Other eating disorders	0.01	0.02	0.04
Females			
10–14 years	0.06	0.12	0.29
15–19 years	0.29	0.32	0.76
20–24 years	0.25	0.36	0.51
25–29 years	0.12	0.25	0.34
30–34 years	0.06	0.15	0.21
35–39 years	0.03	0.08	0.15
40–44 years	0.02	0.05	0.09
45–49 years	0.01	0.02	0.05
50+ years	0.00	0.01	0.01
All ages	0.06	0.09	0.16
Males			
10–14 years	0.01	0.02	0.03
15–19 years	0.01	0.03	0.05
20–24 years	0.00	0.02	0.02
25–29 years	0.00	0.01	0.01
30–34 years	0.00	0.01	0.01
35–39 years	0.00	0.00	0.01
40–44 years	0.00	0.00	0.01
45–49 years	0.00	0.00	0.01
50+ years	0.00	0.00	0.00
All ages	0.00	0.01	0.01

*Note:* Prevalence is calculated as the number of cases diagnosed with an eating disorder in the given year divided by the number of individuals alive and living in Denmark aged 10 years or above on 31st December in the given year.

Abbreviations: AN = anorexia nervosa; BN = bulimia nervosa; OED = other eating disorders.

### 3.4 Somatic Disease and Psychiatric Disorder Comorbidities

#### 3.4.1 ICD‐10 Chapters (1st Level)

As expected, all EDs were most strongly associated with ‘mental and behavioural disorders’, with odds ratios (ORs) ranging from 5.62 for AN to 8.46 for OED (Table 3). The median age difference between the first ED diagnosis and first diagnosis of other mental and behavioural disorders was 0 years (for AN) and approximately 2 for BN and OED. Supporting Information S1: Figures S7–S9 show the associations between each ED and specific psychiatric ICD‐10 codes within the chapter ‘mental and behavioural disorders’.

**TABLE 3:** Association between each eating disorder and ICD10 chapters (1st level) and number of years between ED diagnosis and comorbidity.

ICD 10 chapter	Overall *N*	All events	Male events	Female events	Median age at diagnosis (years)	Median age difference (years)	Odds ratio	Lower 95% CI	Upper 95% CI	*p* value	*q* value	Significant
Anorexia nervosa												
Certain infectious and parasitic diseases	1839669	350048	173098	176950	7.1	−3.05	1.35	1.29	1.4	1.11047395521494E‐43	5.51082786660446E‐39	TRUE
Neoplasms	1839669	105487	41021	64466	19.28	1.61	1.19	1.12	1.27	4.58049326245867E‐08	0.0012123260724851	TRUE
Diseases of the blood and blood‐forming organs and certain disorders involving the immune mechanism	1839669	44239	17936	26303	14.75	0.56	1.83	1.69	1.98	1.54825514225325E‐48	8.78097916657206E‐44	TRUE
Endocrine, nutritional and metabolic diseases	1839669	225391	68765	156626	18.37	0.13	1.53	1.47	1.6	4.27369891185153E‐85	3.39337904451437E‐80	TRUE
Mental and behavioural disorders	1839669	363461	185029	178432	16.22	0	5.62	5.42	5.84	0	0	TRUE
Diseases of the nervous system	1839669	149392	70553	78839	16.05	−0.11	1.31	1.23	1.38	1.29703671410352E‐20	4.29110833533371E‐16	TRUE
Diseases of the eye and adnexa	1839669	163179	83153	80026	13.22	−0.84	1.31	1.24	1.39	4.54018001292629E‐21	1.63862223248414E‐16	TRUE
Diseases of the ear and mastoid process	1839669	173901	93099	80802	4.98	−9.08	1.14	1.07	1.21	3.06755076579434E‐05	0.761149964975597	FALSE
Diseases of the circulatory system	1839669	79398	39081	40317	20.09	0.95	1.88	1.77	2.01	6.79729723349418E‐82	4.49762732222239E‐77	TRUE
Diseases of the respiratory system	1839669	561761	297378	264383	5.15	−7.54	1.18	1.14	1.23	1.14648286164005E‐17	3.50124636025345E‐13	TRUE
Diseases of the digestive system	1839669	417057	208179	208878	14.59	−0.48	1.49	1.44	1.55	4.71725239646332E‐91	6.24261159556386E‐86	TRUE
Diseases of the skin and subcutaneous tissue	1839669	262493	132037	130456	15.56	0	1.33	1.27	1.39	2.39510243121521E‐33	9.50873068878638E‐29	TRUE
Diseases of the musculoskeletal system and connective tissue	1839669	498545	240510	258035	16	−1.08	1.31	1.26	1.36	4.20059481621191E‐43	1.85296296120809E‐38	TRUE
Diseases of the genitourinary system	1839669	367162	150381	216781	19.43	2.23	1.49	1.43	1.55	5.30865915999222E‐86	5.26894065291744E‐81	TRUE
Congenital malformations, deformations and chromosomal abnormalities	1839669	207547	117000	90547	5.65	−5.91	1.22	1.15	1.29	5.0937584793143E‐12	1.44447080670805E‐07	TRUE
Symptoms, signs and abnormal clinical and laboratory findings, not elsewhere classified	1839669	768721	368827	399894	11.9	−1.37	1.83	1.77	1.9	1.80951273983145E‐225	3.59194853145701E‐220	TRUE
Bulimia nervosa												
Certain infectious and parasitic diseases	1839669	350048	173098	176950	7.1	−1.57	1.58	1.49	1.68	2.96600102081192E‐52	2.30155795195938E‐49	TRUE
Neoplasms	1839669	105487	41021	64466	19.28	1.93	1.14	1.04	1.24	0.00307570935808472	0.745840451645181	FALSE
Diseases of the blood and blood‐forming organs and certain disorders involving the immune mechanism + A1:M24	1839669	44239	17936	26303	14.75	0.22	1.48	1.31	1.67	4.29251896511388E‐10	1.28111906191479E‐07	TRUE
Endocrine, nutritional and metabolic diseases	1839669	225391	68765	156626	18.37	0.49	1.49	1.4	1.58	3.15518728862761E‐39	2.04030228555411E‐36	TRUE
Mental and behavioural disorders	1839669	363461	185029	178432	16.22	−0.2	6.67	6.3	7.06	0	0	TRUE
Diseases of the nervous system	1839669	149392	70553	78839	16.05	0	1.37	1.27	1.47	3.09667761739546E‐16	1.0922547265985E‐13	TRUE
Diseases of the eye and adnexa	1839669	163179	83153	80026	13.22	−0.73	1.32	1.22	1.43	1.88699523375913E‐12	6.10112861151752E‐10	TRUE
Diseases of the ear and mastoid process	1839669	173901	93099	80802	4.98	−8.83	1.29	1.19	1.41	5.85043609999437E‐09	1.62136512547774E‐06	TRUE
Diseases of the circulatory system	1839669	79398	39081	40317	20.09	1.14	1.63	1.49	1.78	1.27323912202735E‐26	5.48893499939532E‐24	TRUE
Diseases of the respiratory system	1839669	561761	297378	264383	5.15	−6.61	1.38	1.31	1.46	3.67680492764075E‐30	1.78320480160744E‐27	TRUE
Diseases of the digestive system	1839669	417057	208179	208878	14.59	−1.24	1.56	1.48	1.65	2.25064138162065E‐57	2.18306632926406E‐54	TRUE
Diseases of the skin and subcutaneous tissue	1839669	262493	132037	130456	15.56	−0.59	1.48	1.39	1.58	1.02150618371084E‐34	5.66191809779857E‐32	TRUE
Diseases of the musculoskeletal system and connective tissue	1839669	498545	240510	258035	16	−2.6	1.3	1.24	1.38	4.46461704355248E‐22	1.73222712786335E‐19	TRUE
Diseases of the genitourinary system	1839669	367162	150381	216781	19.43	1.34	1.72	1.63	1.82	5.67703151390726E‐86	7.34210638762161E‐83	TRUE
Congenital malformations, deformations and chromosomal abnormalities	1839669	207547	117000	90547	5.65	−6.13	1.15	1.05	1.25	0.00160208556577393	0.414395538836437	FALSE
Symptoms, signs and abnormal clinical and laboratory findings, not elsewhere classified	1839669	768721	368827	399894	11.9	−2.02	1.87	1.77	1.97	3.66579068660401E‐111	7.11145212507591E‐108	TRUE
Other eating disorders												
Certain infectious and parasitic diseases	1839669	350048	173098	176950	7.1	−3.97	1.51	1.45	1.57	2.5072426461992E‐89	5.0781909413703E‐82	TRUE
Neoplasms	1839669	105487	41021	64466	19.28	0.2	1.19	1.12	1.26	7.51601377093899E‐08	0.761149964975597	FALSE
Diseases of the blood and blood‐forming organs and certain disorders involving the immune mechanism	1839669	44239	17936	26303	14.75	0.25	1.97	1.82	2.13	2.78346121859393E‐66	4.1001064923498E‐59	TRUE
Endocrine, nutritional and metabolic diseases	1839669	225391	68765	156626	18.37	0	1.99	1.91	2.07	5.07327443525543E‐249	2.740121045644E‐241	TRUE
Mental and behavioural disorders	1839669	363461	185029	178432	16.22	−0.18	8.46	8.14	8.8	0	0	TRUE
Diseases of the nervous system	1839669	149392	70553	78839	16.05	−0.78	1.72	1.64	1.81	1.2756872756367E‐100	3.4450507224102E‐93	TRUE
Diseases of the eye and adnexa	1839669	163179	83153	80026	13.22	−2.09	1.45	1.37	1.53	9.17944215453938E‐43	1.14413055600354E‐35	TRUE
Diseases of the ear and mastoid process	1839669	173901	93099	80802	4.98	−9.42	1.38	1.31	1.46	6.07303321655784E‐31	6.56019946878644E‐24	TRUE
Diseases of the circulatory system	1839669	79398	39081	40317	20.09	0.53	1.85	1.73	1.97	2.60854333017394E‐79	4.69632553143759E‐72	TRUE
Diseases of the respiratory system	1839669	561761	297378	264383	5.15	−8.88	1.4	1.35	1.45	2.6057575337961E‐70	4.22217907743086E‐63	TRUE
Diseases of the digestive system	1839669	417057	208179	208878	14.59	−1.56	1.76	1.69	1.82	1.75121795602465E‐192	7.09386398969449E‐185	TRUE
Diseases of the skin and subcutaneous tissue	1839669	262493	132037	130456	15.56	−0.93	1.44	1.38	1.5	7.04797513471171E‐59	9.51668653167727E‐52	TRUE
Diseases of the musculoskeletal system and connective tissue	1839669	498545	240510	258035	16	−2.33	1.47	1.42	1.52	2.77453229888037E‐92	6.42235624658825E‐85	TRUE
Diseases of the genitourinary system	1839669	367162	150381	216781	19.43	0.88	1.59	1.53	1.65	2.45002749211962E‐123	7.93969099748735E‐116	TRUE
Congenital malformations, deformations and chromosomal abnormalities	1839669	207547	117000	90547	5.65	−7.27	1.37	1.3	1.44	1.57543055840729E‐31	1.82336610244834E‐24	TRUE
Symptoms, signs and abnormal clinical and laboratory findings, not elsewhere classified	1839669	768721	368827	399894	11.9	−2.71	2.17	2.09	2.25	0	0	TRUE

*Note:* ‘Median age difference (years)’ indicates that half or more of the individuals with EDs and a given comorbidity were diagnosed with the comorbidity before the ED diagnosis (negative value) or after the ED diagnosis (positive value).

Abbreviation: ICD‐10 = International Classification of Diseases version 10.

In relation to disorders affecting other systems, AN was most strongly associated with ‘diseases of the circulatory system’ (OR = 1.88, 95% CI 1.77, 2.01, *q* < 0.001), with diagnoses occurring almost 1 year after AN, suggesting these might be a consequence of the ED. ‘Symptoms, signs and abnormal clinical findings’ showed the greatest association with BN (OR = 1.87, 95% CI 1.77, 1.97, *q* < 0.001) and OED (OR = 2.17, 95% CI 2.09, 2.25, *q* < 0.001), with diagnoses occurring 2 and 2.71 years before the ED diagnosis, respectively. This suggests individuals with a disorder other than AN might present to clinical services with blood test abnormalities and not be recognised as having an ED.

#### 3.4.2 ICD‐10 Sub‐categories (2nd Level)

**FIGURE 3:**
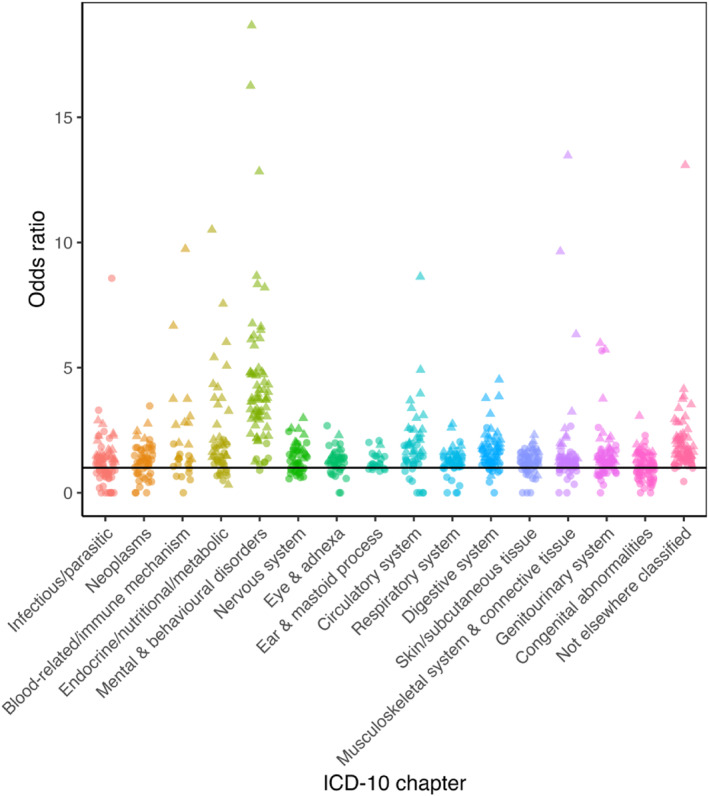
Association between anorexia nervosa and diseases in each of the International Classification of Diseases version 10 (ICD‐10) chapters. Significant results are indicated by a triangle whilst non‐significant are circles. For ease of interpretation, odds ratios above 20 have been excluded (*N* = 3 in ICD‐10 chapters Endocrine/nutritional/metabolic and Nervous system). ICD‐10 chapters XV (Pregnancy, childbirth and the puerperium) and XVI (Certain conditions originating in the perinatal period) are not included.

**FIGURE 4:**
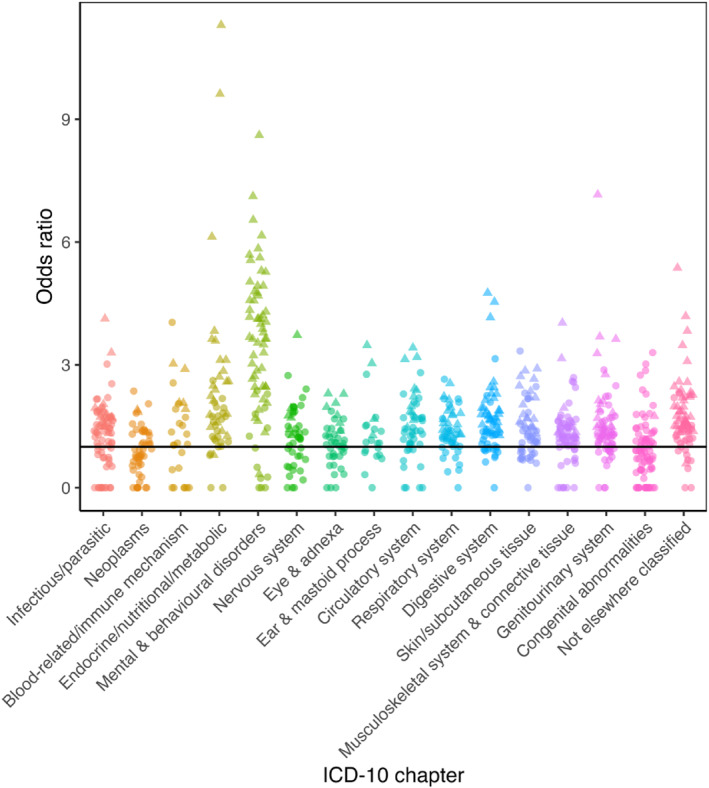
Association between bulimia nervosa and diseases in each of the International Classification of Diseases version 10 (ICD‐10) chapters. Significant results are indicated by a triangle whilst non‐significant are circles. For ease of interpretation, odds ratios above 20 have been excluded (*N* = 6 in ICD‐10 chapters Infectious/parasitic, Endocrine/nutritional/metabolic, Mental & behavioural disorders, Nervous system, and Digestive system). ICD‐10 chapters XV (Pregnancy, childbirth and the puerperium) and XVI (Certain conditions originating in the perinatal period) are not included.

**FIGURE 5:**
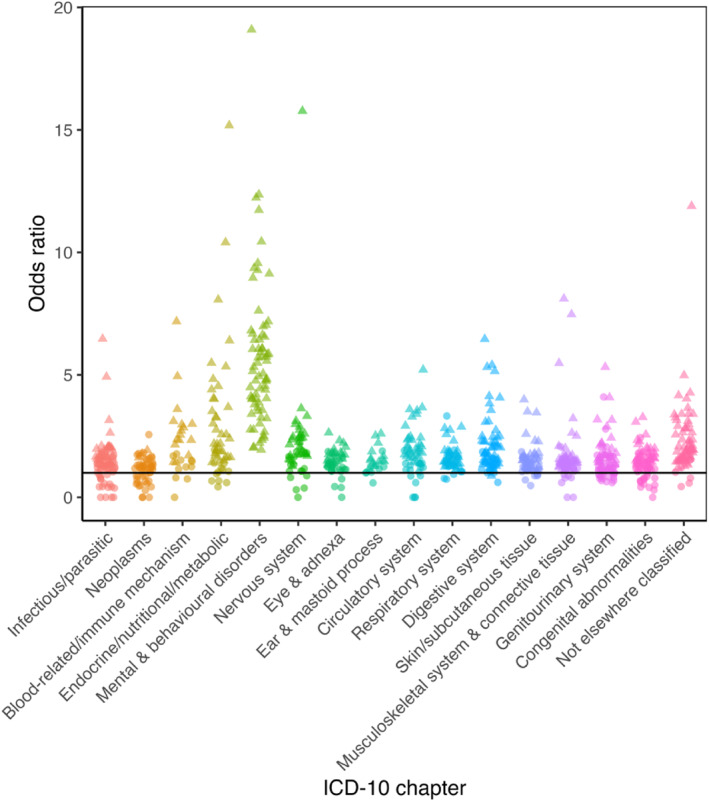
Association between other eating disorders and diseases in each of the International Classification of Diseases version 10 (ICD‐10) chapters. Significant results are indicated by a triangle whilst non‐significant are circles. For ease of interpretation, odds ratios above 20 have been excluded (*N* = 1 in ICD‐10 chapter Endocrine/nutritional/metabolic). ICD‐10 chapters XV (Pregnancy, childbirth and the puerperium) and XVI (Certain conditions originating in the perinatal period) are not included.

All associations are shown in Supporting Information S2: Tables S4–S6 and Figures 3, 4, 5.

Both AN and OED showed strong associations with ‘nutritional marasmus’ (i.e., severe protein‐calorie malnutrition) (OR = 41.89, 95% CI 28.88, 60.76, *q* < 0.001 and OR = 39.47, 95% CI 27.15, 57.39, *q* < 0.001, respectively). For half of the participants, this was at least 4.9 months subsequent to AN and at least 1 year subsequent to an OED diagnosis, suggesting this as an effect of the ED. Aligned with this finding, AN also showed strong associations with a subsequent ‘dietary zinc deficiency’ diagnosis (OR = 38.01, 95% CI 25.79, 56.01, *q* < 0.001, a median of 2.4 months after AN diagnosis). ‘Polyneuropathy in diseases classified elsewhere’ was strongly associated with AN (OR = 21.51, 95% CI 5.29, 87.55, *q* < 0.001), BN (OR = 34.72, 95% CI 4.8, 251.21, *q* < 0.01), and OED (OR = 15.77, 95% CI 3.88, 64.1, *q* < 0.001), with diagnoses of this comorbidity occurring at least 7.55 years, 10.96 years, and 6.58 years before each ED diagnosis for half of the cases, respectively. OED was associated with a subsequent ‘abuse of non‐dependence‐producing substances’ (e.g., laxatives, analgesics, etc; OR = 19.09, 95% CI 14.89, 24.48, *q* < 0.001), with diagnoses occurring with a median of 1.5 years after OED diagnosis. BN was associated with subsequent ‘psychological and behavioural disorders associated with sexual development and orientation’ (OR = 32.55, 95% CI 4.51, 235.04, *q* < 0.01) and ‘alcoholic liver disease’ (OR = 26.92, 95% CI 3.72, 194.69, *q* < 0.01), with diagnoses recorded a median of 2.8 months and 5.9 years after BN diagnosis, respectively.

#### 3.4.3 Prescriptions

All three ED diagnoses were associated with prescription of several psychotropics and other medications (Table 4). Below we report the strongest associations.

**TABLE 4:** Association between each eating disorder and ATC chapters (1st level) and number of years between ED diagnosis and comorbidity.

ATC group	Overall *N*	All events	Male events	Female events	Median age at diagnosis (years)	Median age difference (years)	Odds ratio	Lower 95% CI	Upper 95% CI	*p* value	*q* value	Significant
Anorexia nervosa												
Alimentary tract/metabolism	1839669	849402	387931	461471	11.83	−2.15	1.57	1.52	1.64	5.28989015902005E‐121	4.06099520381632E‐120	TRUE
Blood/blood‐forming organs	1839669	111316	25239	86077	22.21	2.87	1.42	1.35	1.5	2.3524615931888E‐38	1.20397399062713E‐37	TRUE
Cardiovascular system	1839669	358597	145383	213214	21.59	2.87	1.22	1.17	1.27	5.05703188440064E‐20	1.94111613008441E‐19	TRUE
Dermatologicals	1839669	1599380	806098	793282	5.18	−11.13	1.35	1.26	1.45	6.92747769262203E‐17	2.12726184012064E‐16	TRUE
Genitourinary system/sex hormones	1839669	639268	35918	603350	16.55	−0.4	0.87	0.82	0.92	2.48429061758602E‐07	3.46757235530255E‐07	TRUE
Systemic hormonal	1839669	262031	132522	129509	13.36	0.28	1.15	1.1	1.21	1.00009003298821E‐08	1.5355180184316E‐08	TRUE
Anti‐infectives	1839669	1760415	898662	861753	2.37	−12.98	1.58	1.37	1.83	4.98744132434263E‐10	1.09394522589929E‐09	TRUE
Antineoplastic/immunomodulating agents	1839669	32013	8648	23365	24.54	4.57	1.15	1.03	1.27	0.00948957994493093	0.0112077763094758	TRUE
Musculoskeletal system	1839669	761515	346670	414845	18.44	0.07	1.06	1.02	1.11	0.00249664075815241	0.00319440978915256	TRUE
Nervous system	1839669	836142	397026	439116	18.76	0.06	2.56	2.46	2.67	0	0	TRUE
Antiparasitic/insecticides/repellents	1839669	809938	345900	464038	10.67	−4.35	1.12	1.08	1.16	8.79698004871944E‐10	1.68833816544385E‐09	TRUE
Respiratory system	1839669	1342652	681647	661005	3.63	−11.41	1.15	1.1	1.2	1.37323931017252E‐09	2.34271541653995E‐09	TRUE
Sensory organs	1839669	1546757	785591	761166	1.99	−12.65	1.25	1.18	1.32	2.65608873201757E‐15	6.79684157098646E‐15	TRUE
Various	1839669	41271	25047	16224	13.1	−2.54	0.98	0.86	1.12	0.793184824346321	0.869885674257826	FALSE
Bulimia nervosa												
Alimentary tract/metabolism	1839669	849402	387931	461471	11.83	−2.51	1.99	1.87	2.11	3.15182577187393E‐115	2.80200995635399E‐114	TRUE
Blood/blood‐forming organs	1839669	111316	25239	86077	22.21	1.91	1.38	1.29	1.48	3.80407013730075E‐20	7.51525071560046E‐20	TRUE
Cardiovascular system	1839669	358597	145383	213214	21.59	1.24	1.41	1.33	1.49	1.01941175152052E‐33	4.53134482063466E‐33	TRUE
Dermatologicals	1839669	1599380	806098	793282	5.18	−12.32	1.98	1.74	2.26	1.92189801006925E‐24	6.83435919252501E‐24	TRUE
Genitourinary system/sex hormones	1839669	639268	35918	603350	16.55	−3.92	1.78	1.59	1.99	3.94181934028424E‐23	1.00123527822128E‐22	TRUE
Systemic hormonal	1839669	262031	132522	129509	13.36	−0.16	1.32	1.24	1.41	1.75162165494313E‐18	3.11442425575306E‐18	TRUE
Anti‐infectives	1839669	1760415	898662	861753	2.37	−14.83	5.08	3.31	7.79	9.351070400753E‐14	1.38553515020273E‐13	TRUE
Antineoplastic/immunomodulating agents	1839669	32013	8648	23365	24.54	5.44	0.97	0.85	1.11	0.671163684303308	0.852389087594491	FALSE
Musculoskeletal system	1839669	761515	346670	414845	18.44	−2.16	1.34	1.26	1.42	6.41762679404294E‐21	1.42633631065059E‐20	TRUE
Nervous system	1839669	836142	397026	439116	18.76	−0.92	3.3	3.07	3.55	7.586942945202E‐226	1.34897619408107E‐224	TRUE
Antiparasitic/insecticides/repellents	1839669	809938	345900	464038	10.67	−3.78	1.56	1.48	1.65	2.58724090675077E‐56	1.53339160747069E‐55	TRUE
Respiratory system	1839669	1342652	681647	661005	3.63	−11.8	1.44	1.34	1.54	2.43355450784355E‐24	7.21152801989388E‐24	TRUE
Sensory organs	1839669	1546757	785591	761166	1.99	−13.38	1.42	1.31	1.54	3.66911374736615E‐18	5.93070002493577E‐18	TRUE
Various	1839669	41271	25047	16224	13.1	−4.11	1.09	0.91	1.31	0.355300789949262	0.485948547333253	FALSE
Other eating disorders												
Alimentary tract/metabolism	1839669	849402	387931	461471	11.83	−4.1	1.99	1.92	2.07	2.03E‐264	9.98E‐263	TRUE
Blood/blood‐forming organs	1839669	111316	25239	86077	22.21	1.54	1.71	1.63	1.8	4.16E‐101	1.36E‐99	TRUE
Cardiovascular system	1839669	358597	145383	213214	21.59	0.91	1.37	1.31	1.42	3.89E‐52	9.56E‐51	TRUE
Dermatologicals	1839669	1599380	806098	793282	5.18	−12.11	1.45	1.35	1.56	8.79E‐24	8.65E‐23	TRUE
Genitourinary system/sex hormones	1839669	639268	35918	603350	16.55	−1.88	1.04	0.99	1.1	0.14224275	1	FALSE
Systemic hormonal	1839669	262031	132522	129509	13.36	−1.81	1.33	1.27	1.39	1.72E‐35	2.42E‐34	TRUE
Anti‐infectives	1839669	1760415	898662	861753	2.37	−13.87	1.88	1.61	2.2	3.78E‐15	3.38E‐14	TRUE
Antineoplastic/immunomodulating agents	1839669	32013	8648	23365	24.54	3.51	1.08	0.98	1.2	0.12924003	0.97847921	FALSE
Musculoskeletal system	1839669	761515	346670	414845	18.44	−1.36	1.35	1.29	1.4	1.62E‐49	2.66E‐48	TRUE
Nervous system	1839669	836142	397026	439116	18.76	−0.76	3.33	3.19	3.49	0	0	TRUE
Antiparasitic/insecticides/repellents	1839669	809938	345900	464038	10.67	−4.75	1.21	1.17	1.26	2.83E‐25	3.10E‐24	TRUE
Respiratory system	1839669	1342652	681647	661005	3.63	−12.22	1.43	1.37	1.5	7.82E‐51	1.54E‐49	TRUE
Sensory organs	1839669	1546757	785591	761166	1.99	−13.29	1.37	1.29	1.45	2.79E‐27	3.43E‐26	TRUE
Various	1839669	41271	25047	16224	13.1	−4.01	1.14	1.01	1.28	0.03185808	0.26129806	FALSE

*Note:* ‘Median age difference (years)’ indicates that half or more of the individuals with EDs and a given comorbidity were diagnosed with the comorbidity before the ED diagnosis (negative value) or after the ED diagnosis (positive value).

Abbreviation: ATC = Anatomical Therapeutic Chemical classification.

#### 3.4.4 Anatomical or Pharmacological Group Medication (1st Level)

Diagnosis of AN showed high levels of prescribed medications related to the ‘nervous system’ group (OR = 2.56, 95% CI 2.46, 2.67, *q* < 0.001), with prescription occurring with a median of < 1 month after AN diagnosis (Supporting Information S1: Figure S10). This was also true for OED (OR = 3.33, 95% CI 3.19, 3.49, *q* < 0.001), albeit in this case prescription preceded the OED diagnosis (∼9 months prior) (Supporting Information S1: Figure S11). BN showed the strongest association with prescription medications within the ‘anti‐infectives for systemic use’ group (OR = 5.08, 95% CI 3.31, 7.79, *q* < 0.001), with prescription preceding the BN diagnosis by 14.8 years (Supporting Information S1: Figure S12).

#### 3.4.5 Pharmacological or Therapeutic Sub‐Categories Medication (2nd Level)

Here, we report the results with the top three highest ORs for each ED and ATC code for pharmacological or therapeutic ATC sub‐categories (Supporting Information S2: Tables S7–S9 and Supporting Information S1: Figures S10–S12). In line with the 1st level prescriptions but more specifically psychostimulant prescription (i.e., substances that increase activity in the central nervous system) was strongly associated with all three EDs: AN (OR = 4.68, 95% CI 4.5, 4.86, *q* < 0.001), BN (OR = 5.61, 95% CI 5.31, 5.93, *q* < 0.001), and OED (OR = 5.58, 95% CI 5.38, 5.79, *q* < 0.001), with prescriptions occurring before and after the ED diagnosis (4.4 months after an AN diagnosis, 2 months before a BN diagnosis, and contemporaneous to an OED diagnosis). ‘Mineral supplements’ prescription following the ED diagnosis was strongly associated with all three EDs: AN (OR 8.33, 95% CI 7.76, 8.94, *q* < 0.001), BN (OR = 6.48, 95% CI 5.88, 7.15, *q* < 0.001), and OED (OR = 7.80, 95% CI 7.26, 8.37, *q* < 0.001). A diagnosis of AN was also linked with higher odds of being prescribed ‘drugs for treatment of bone diseases’ (OR = 6.06, 95% CI 4.33, 8.48, *q* < 0.001), a median of 5.4 years after AN diagnosis‐reflecting high levels of bone loss and osteoporosis as a consequence of AN. Interestingly, BN was associated with prescriptions of ‘antibacterials for systemic use’ (OR = 4.11, 95% CI 2.89, 5.85, *q* < 0.001), a median of 14.8 years before BN diagnosis, potentially highlighting infections as more common in early life in this group of individuals. OED was associated with ‘anti‐Parkinson drugs’ (OR = 4.83, 95% CI 4.31, 5.42, *q* < 0.001) with a median of 2 years after OED diagnosis.

#### 3.4.6 Chemical, Pharmacological, or Therapeutic Subgroup (3rd Level)

Diagnoses of AN, BN, and OED were all significantly associated with prescriptions of antidepressants and anxiolytics (Supporting Information S2: Table S10), with ORs ranging from 2.54 to 2.99 for anxiolytics and 5.46 to 6.32 for antidepressants.

## 4 Discussion

This is the first study to use Danish registers up to 2022 to explore the sex‐and‐age‐stratified epidemiology of AN, BN, and OED over 22 years, focusing on incidence during the COVID‐19 pandemic. Similar to earlier findings in Denmark (Momen et al. 2024; H.‐C. Steinhausen and Jensen 2015; Zerwas et al. 2015) and other countries (Cybulski et al. 2021; Trafford et al. 2023), ED incidence increased over time, especially for AN and OED in younger age groups (< 20 years for AN, < 25 for OED). BN diagnoses, however, have declined since 2017, potentially due to recognition of binge‐eating disorder (BED) in DSM‐5 (2013) and ICD‐11 (2022). These changes may have led to increased recognition of BED cases, and therefore BED cases to be classified under OED rather than BN, which aligns with our observed rise in OED incidence and decrease in the incidence of BN. The largest increases were seen in AN incidence for females aged 10–19 (doubling since 2005) and males aged 10–14, alongside rising OED incidence in youth (10–19 years), potentially reflecting recognition of other EDs, like BED and Avoidant and Restrictive Food Intake Disorder. Time trends in young females' AN incidence were highlighted in a recent review (van Eeden et al. 2021); a UK‐based study found an increase in ED incidence in young females between 2003 and 2018, with nearly a doubling of rates for girls aged 13–16 (Cybulski et al. 2021). Our rates are comparable (25 per 10,000 PY). This increase pre‐dates the COVID period and might be due to increased recognition. While the observed rise in ED incidence may reflect genuine increases, potentially driven by heightened environmental pressures such as social media or the trend towards earlier onset of puberty (Cheng et al. 2022), it may also be influenced by shifts in diagnostic practices, clinical awareness, stigma, or healthcare access. Further investigation is needed to better understand the underlying drivers of this trend.

We observed an important increase in ED incidence in young males aged 10–19 (doubling/trebling) over the last 22 years, with rates for males aged 10–14 reaching ∼3 per 10,000 PY in 2022, similar to the UK in 2018 (Cybulski et al. 2021). Overall, ED incidence in males has steadily increased in Denmark since the early 2000s, particularly for those below the age of 20, suggesting underdiagnosis of males earlier in the century and raising hopes for a gradual improvement in recognising EDs in males. Despite this, the male‐to‐female ratio for ED incidence remains lower than the widely reported 1:10 ratio (Carlat et al. 1997), indicating ongoing underdiagnosis in males. As discussed in a Norwegian study (Reas and Rø 2018), the gap between expected and observed male‐to‐female ratios is highest for BN (up to ∼40/45:1 in our data). This has important public health implications, pointing to a likely treatment gap for males with EDs, underscoring the need for better training of healthcare professionals and public awareness. This is particularly important given the evidence from Danish registers of high mortality in males with EDs (Micali and Herle 2023).

Given that IRs in older age groups did not decrease, our findings are more likely to indicate increased detection or an increase in the number of individuals developing an ED rather than a shift in age at diagnosis or earlier detection. Whilst this is true for incidence of overall EDs, we did observe a decrease in new diagnoses of BN around 2016/2017 and an increase in new diagnoses of OED in 2017/2018. Danish healthcare might not be prepared for this incidence increase, this is corroborated by a slow adoption of ICD‐11 (not adopted yet), inadvertently delaying access to assessment and treatment to individuals who might meet ED diagnoses (e.g., individuals with BED or ARFID).

Our study is one of the first studies to leverage national registers to study the sex‐and‐age stratified incidence of EDs during the COVID‐19 pandemic. The incidence of ED diagnoses increased during the COVID‐19 pandemic compared to pre‐pandemic, consistent with previous literature in Denmark (Sonne et al. 2024); this is particularly true for females aged 10–14, who show a peak in new diagnoses in 2021. New ED diagnoses slightly decrease in 2022 in this age group, however, ED incidence rates did not quite go back to pre‐pandemic levels. The pandemic increase in ED diagnoses is mostly due to an increase in incidence of AN and OED in females below the age of 20 years. Our findings and incidence estimates are also consistent with those of a study of electronic healthcare records (mostly including US care providers), although this study focused on a short temporal window (January 2020 to January 2021) (Taquet et al. 2021).

### 4.1 Impact of EDs

Prevalence of EDs increased steadily from 2000 to 2010 to 2022, but this trend was driven by females. Research with community samples has shown that males do indeed have EDs (Flament et al. 2015) but our study demonstrates that these are under‐detected in the clinic.

As expected, all EDs were most strongly associated with the ICD‐10 chapter ‘mental and behavioural disorders’. Within this category, AN and OED were most strongly associated with abuse of non‐dependence‐producing substances, which may reflect laxative use in the binge‐purge subtype of AN or misuse of substances like antidepressants or painkillers. BN was most strongly associated with ‘psychological and behavioural disorders associated with sexual development and orientation’; delayed psychosexual development has been long established in those with EDs (Dunkley et al. 2020; U. Schmidt et al. 1995) and being gender or sexually diverse is linked to increased risk of EDs (Grammer et al. 2021). Of the three EDs, AN was most strongly associated with obsessive‐compulsive disorder, consistent with literature on their high co‐occurrence (Mandelli et al. 2020) and shared aetiology (Meijsen et al. 2024; Yilmaz et al. 2020). All EDs showed strong associations with both antidepressant and anxiolytic prescriptions, with the maximum median time difference in diagnosis and prescription being 1.2 years. This temporal proximity supports the idea that internalising disorders strongly co‐occur with EDs and their relationship is bidirectional.

In addition to the high co‐occurrence with psychiatric illness, all EDs were highly comorbid with somatic diseases. Interesting differences were seen between diagnoses that preceded the ED diagnosis and those likely subsequent effects of the ED. Those with AN had high odds of being prescribed ‘drugs for treatment of bone diseases’ about 5 years after AN diagnosis, as low bone mineral density and osteoporosis are established consequences of AN (Misra et al. 2016). Alcoholic liver disease had high odds of occurring after BN, a median of 6 years after diagnosis, consistent with previous literature (Mellentin et al. 2022) and our finding that BN was most strongly associated with ‘mental and behavioural disorders due to use of alcohol’. Shared risk factors like high impulsivity and difficulties regulating emotions (Azevedo et al. 2021) may explain this co‐occurrence. Several comorbidities reflected the systemic effects of EDs, for example, secondary to poor nutrition, a distinctive characteristic of EDs. For instance, severe malnourishment in the form of ‘nutritional marasmus’ was highly comorbid with AN and OED, and all three EDs were associated with high odds of being prescribed mineral supplements, occurring less than 1 year after ED diagnosis. Infectious and parasitic diseases seemed to occur at higher levels in individuals with EDs, and to occur prior to ED diagnosis, which is in line with a primary care register‐based study from Wales (Demmler et al. 2020) and may lend weight to hypotheses that infections trigger ED onset (Ajueze et al. 2018; Khimani et al. 2024). ‘Polyneuropathy in diseases classified elsewhere’ had high odds of preceding all EDs. This diagnosis typically refers to peripheral nerve damage affecting multiple body regions and is often used for individuals presenting with pain. This aligns with findings from a retrospective, longitudinal study in Denmark, which showed that individuals with chronic pain are more likely to experience depression and anxiety than the general population (Søndergård et al. 2018). Indeed, chronic pain is associated with unemployment, family strain, and a diminished sense of self (Sjøgren et al. 2009), all of which may contribute to the onset of mental health conditions.

Overall, our findings regarding comorbidities reflect previous literature, in that EDs often co‐occur with both psychiatric disorders and somatic diseases, underscoring the severity of EDs and their complex interactions with other illnesses, and providing interesting avenues about shared aetiology to pursue. Future work will aim to delve deeper into these complex interactions.

## 5 Strengths

Our study leveraged a large representative cohort of patients with EDs treated in the Danish healthcare system from the age of 10 years over 22 years. To our knowledge, this is the most extensive nationwide register‐based study looking at trends in incidence of ED during COVID‐19. The inclusion of all Danish citizens via the use of Danish register data means we avoid biases related to the self‐selection of participants. Our inclusion of OED marks an important shift in our recognition of EDs beyond AN and BN and is a core strength of our study. Further, our broad approach to mapping comorbidities (including any diagnoses as well as prescription medication) enabled us to capture cases of milder comorbid illnesses that may not have received a hospital‐based diagnosis. Including males and females allows us to paint a larger and finer map of the epidemiology of EDs, often problematic due to the low number of males.

## 6 Limitations

We only included individuals aged 10 or above, however ED onset before 10 is extremely rare, and any ED coded before this age is likely misclassified or indicates a different disorder. The validity of the ICD‐10 diagnoses registered in the DNPR is unclear. However, a Swedish study found moderate to excellent agreement between ICD‐10 ED diagnoses in the Swedish National Patient Register and the national quality registers (Birgegård et al. 2022) and the validity of the Danish healthcare registers has been demonstrated in EDs (Egedal et al. 2024). Additionally, the pandemic may have affected ED diagnoses due to difficulties accessing hospitals, with increases in 2021 potentially reflecting delayed diagnoses of disorders that began before the pandemic. However, Denmark only had minor restrictions and lockdowns during the pandemic, as compared with some other countries. We only included in‐ and outpatients treated in Danish hospitals. While these are likely representative of those who seek healthcare, our study does not capture people with milder symptoms or those unable or resistant to accessing care, likely leading to lower incidence and prevalence rates than community samples. Therefore, our study likely underestimates the true prevalence of EDs in the total population.

## 7 Conclusions

EDs (specifically AN and OED diagnoses) have increased in incidence in Denmark between 2000 and 2022. Moreover, EDs have high impact and burden on the healthcare system, given the high levels of comorbidities identified and their increasing prevalence. We confirm that the COVID‐19 pandemic and its associated stressors are likely to have resulted in increases in ED cases, particularly in young females with AN and OED. Although it is unclear what led to this increase in incidence, it is likely that clarification of the environmental factor(s) that pushed young people at risk into developing EDs can lead to improved insights into risk factors for these disorders. Service provision will need to adequately match this increased demand. Our study also highlights that whilst there are recent improvements in the identification of male ED cases, clinical recognition and diagnoses of EDs in males should be a priority to achieve equity in healthcare across the sexes. Efforts should be made to improve detection at a primary and secondary healthcare level for males with EDs.

## Funding

This study was funded by a Laureate Grant Award from the Novo Nordisk Foundation (Grant NNF22OC0071010) to N.M. L.H.M and E.R.H were supported in part by grants from the Novo Nordisk Foundation (Grants NNF17OC0027812, NNF17OC0027594).

## Ethics Statement

The study was conducted according to the rules of the Danish Data Protection Agency and did not require any ethical approval or participant consent. Completion of the study was approved by Research law, Mental Health Services of the Capital Region of Denmark, no. p‐2024‐15760.

## Conflicts of Interest

The authors declare no conflicts of interest.

## Permission to Reproduce Material From Other Sources

The authors have nothing to report.

## Supporting information


Supporting Information S1

Supporting Information S2

Supporting Information S3

## Data Availability

Data supporting the results of this study are available from Statistics Denmark. Data are not publicly available but can be accessed by researchers affiliated with an authorised Danish research institution via Statistics Denmark.
