# Supplementary material for: Temporal Trends in the Epidemiology of Eating Disorders Between 2000 and 2022: A Danish Register Study of Their Incidence and Comorbidities
Source: Eur Eat Disord Rev. 2025 Dec 16;34(3):749–69. doi: 10.1002/erv.70061 (PMC13048747; doi:10.1002/erv.70061)
Supplement: Supplementary file 1 — Supporting Information S1 [file ERV-34-749-s003.docx]

**Figure S1.** Female (top) and male (bottom) incidence rate per 10,000 person-years (PY) of other eating disorders (OED) in the years 2000 to 2022, stratified by age groups (10-14 years; 15-19 years; 20-24 years; 25-29 years; 30-34 years). Incidence is calculated via the number of new events in the given calendar year divided by the number of PY per 10,000 amongst the population at risk, individuals aged ≥10 years on 1 January in the given year who had not previously been diagnosed with an eating disorder.

**Figure S2.** Female (top) and male (bottom) incidence rate per 10,000 person-years (PY) of anorexia nervosa in the years 2000 to 2022, stratified by age groups (10-14 years; 15-19 years; 20-24 years; 25-29 years; 30-34 years). Incidence is calculated via the number of new events in the given calendar year divided by the number of PY per 10,000 amongst the population at risk, individuals aged ≥10 years on 1 January in the given year who had not previously been diagnosed with an eating disorder.

**Figure S3.** Female (top) and male (bottom) incidence rate per 10,000 person-years (PY) of bulimia nervosa in the years 2000 to 2022, stratified by age groups (10-14 years; 15-19 years; 20-24 years; 25-29 years; 30-34 years). Incidence is calculated via the number of new events in the given calendar year divided by the number of PY per 10,000 amongst the population at risk, individuals aged ≥10 years on 1 January in the given year who had not previously been diagnosed with an eating disorder.

**Figure S4.** Female (top) and male (bottom) prevalence of anorexia nervosa in the years 2000, 2010, and 2022, stratified by age groups (10-14 years; 15-19 years; 20-24 years; 25-29 years; 30-34 years). Prevalence is calculated as the number of cases diagnosed with an eating disorder in the given year divided by the number of individuals alive and living in Denmark aged 10 years or above on 31st December in the given year.

**Figure S5.** Female (top) and male (bottom) prevalence of other eating disorders (OED) in the years 2000, 2010, and 2022, stratified by age groups (10-14 years; 15-19 years; 20-24 years; 25-29 years; 30-34 years). Prevalence is calculated as the number of cases diagnosed with an eating disorder in the given year divided by the number of individuals alive and living in Denmark aged 10 years or above on 31st December in the given year.

**Figure S6.** Female (top) and male (bottom) prevalence of bulimia nervosa in the years 2000, 2010, and 2022, stratified by age groups (10-14 years; 15-19 years; 20-24 years; 25-29 years; 30-34 years). Prevalence is calculated as the number of cases diagnosed with an eating disorder in the given year divided by the number of individuals alive and living in Denmark aged 10 years or above on 31st December in the given year.

**
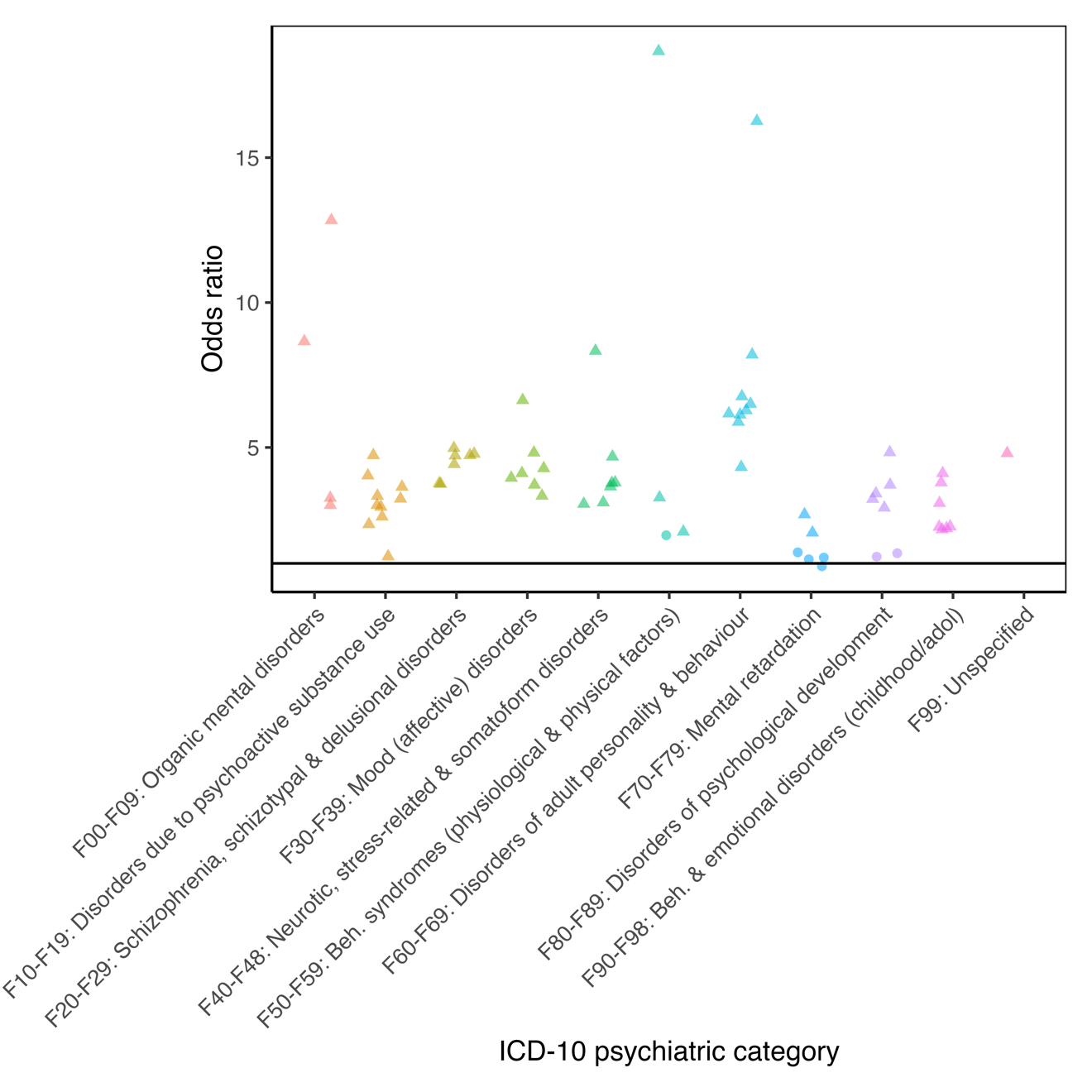
**

**Figure S7.** Association between anorexia nervosa and diagnosis codes within the International Classification of Diseases version 10 (ICD-10) chapter V ‘Mental and behavioural disorders’. *Note.* Significant results are indicated by a triangle whilst non-significant are circles.

**
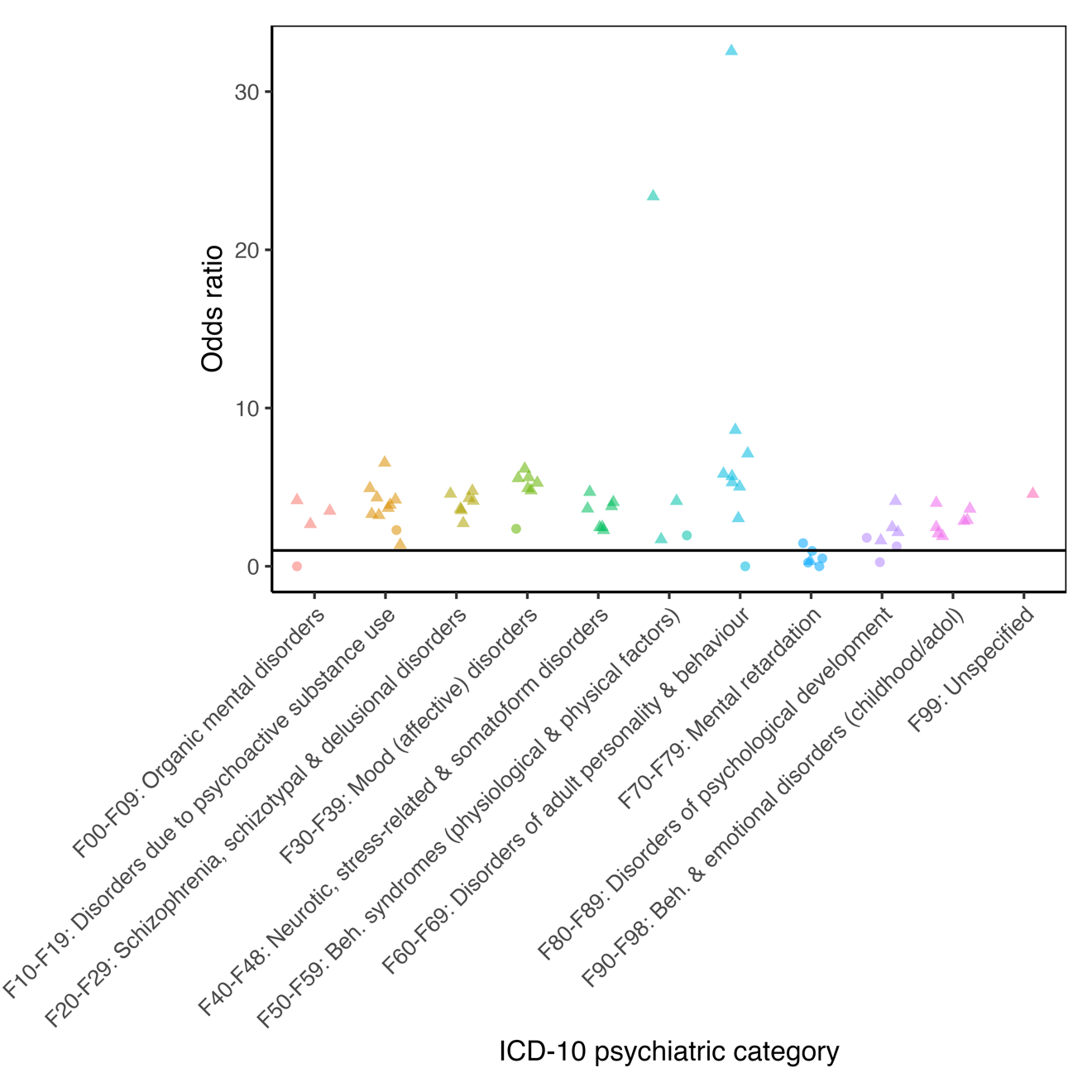
**

**Figure S8.** Association between bulimia nervosa and diagnosis codes within the International Classification of Diseases version 10 (ICD-10) chapter V ‘Mental and behavioural disorders’. *Note.* Significant results are indicated by a triangle whilst non-significant are circles.

**
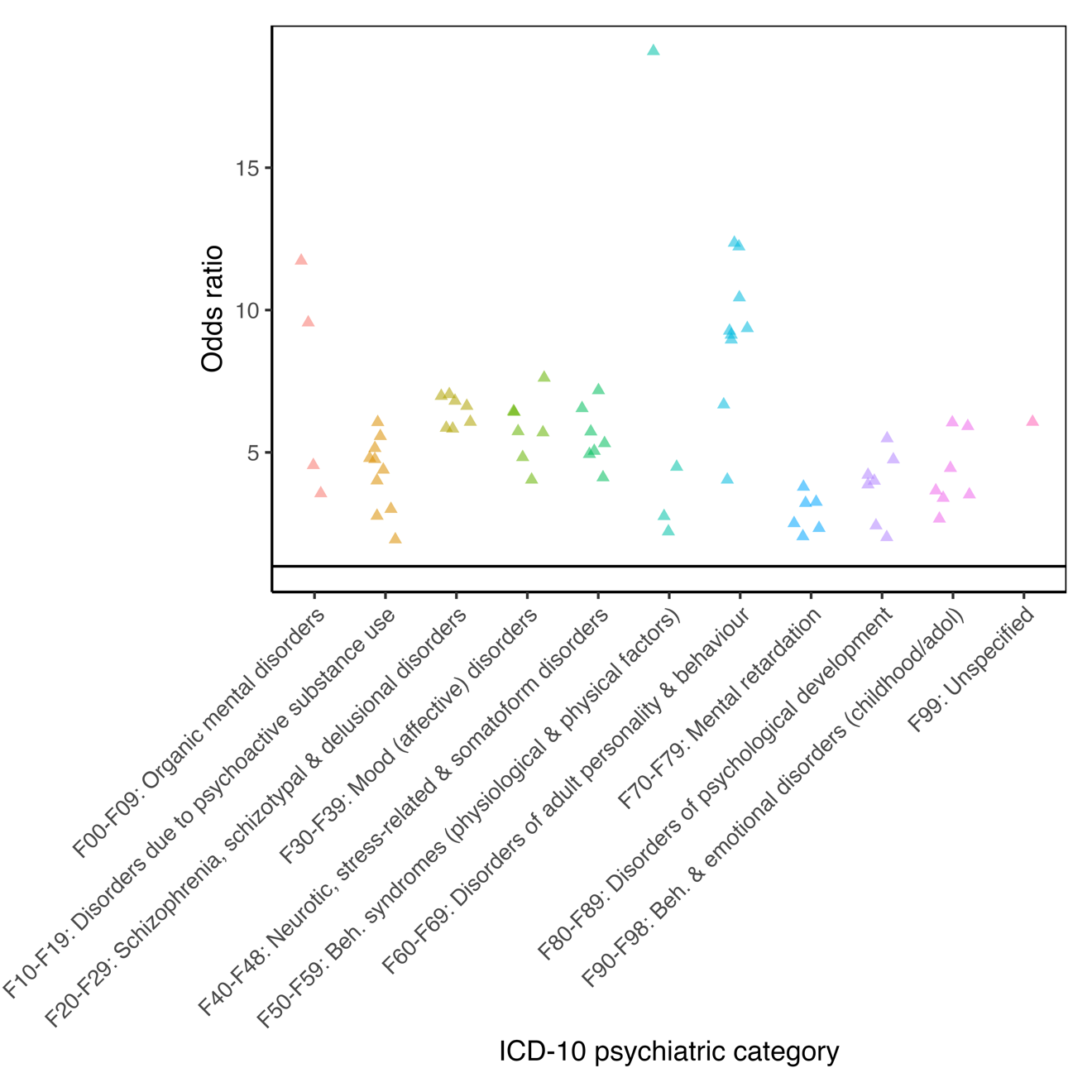
**

**Figure S9.** Association between other eating disorders and diagnosis codes within the International Classification of Diseases version 10 (ICD-10) chapter V ‘Mental and behavioural disorders’. *Note.* Significant results are indicated by a triangle whilst non-significant are circles (all significant).

**
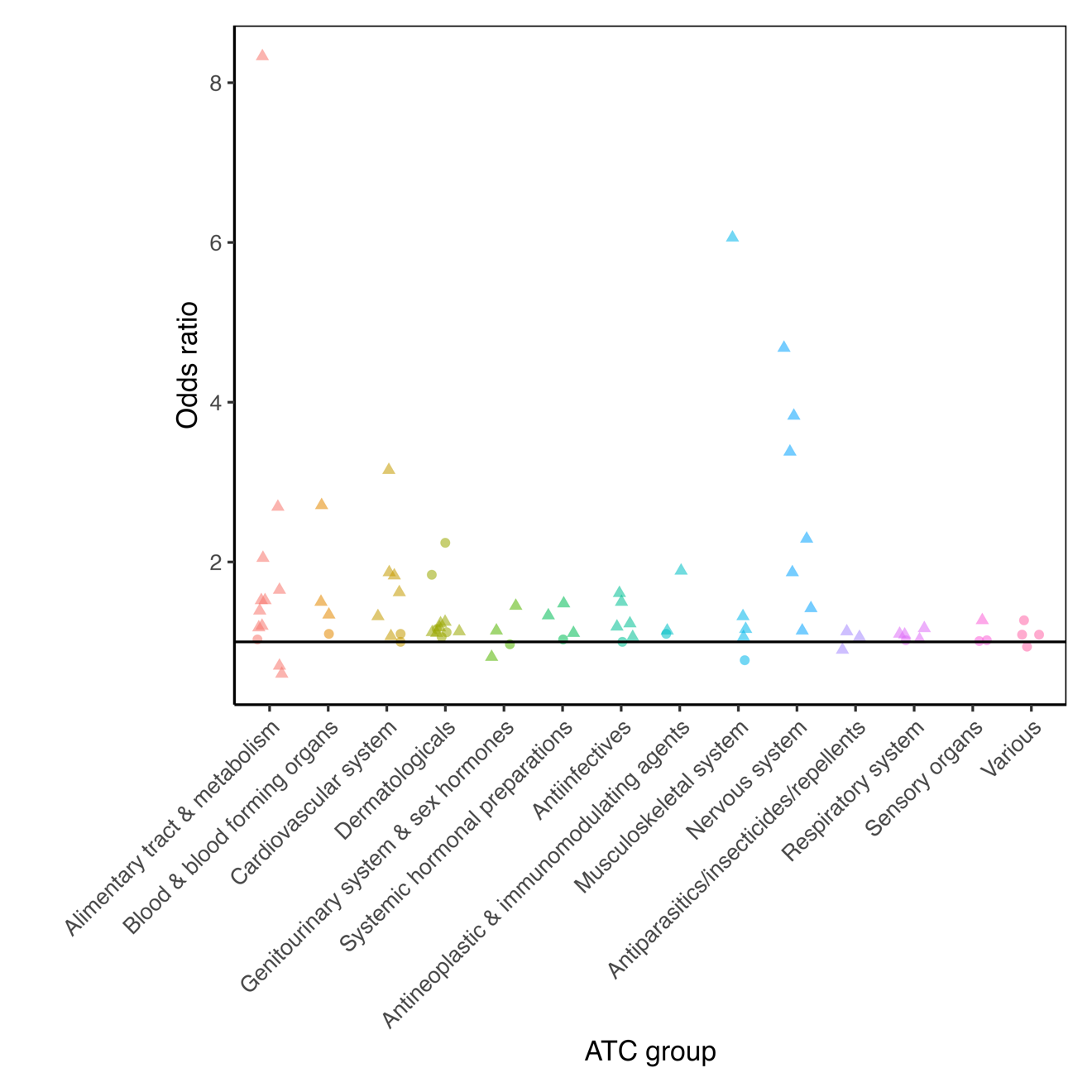
**

**Figure S10.** Association between anorexia nervosa and prescription medication in each of the Anatomical Therapeutic Chemical classification (ATC) chapters. *Note.* Significant results are indicated by a triangle whilst non-significant are circles.

**
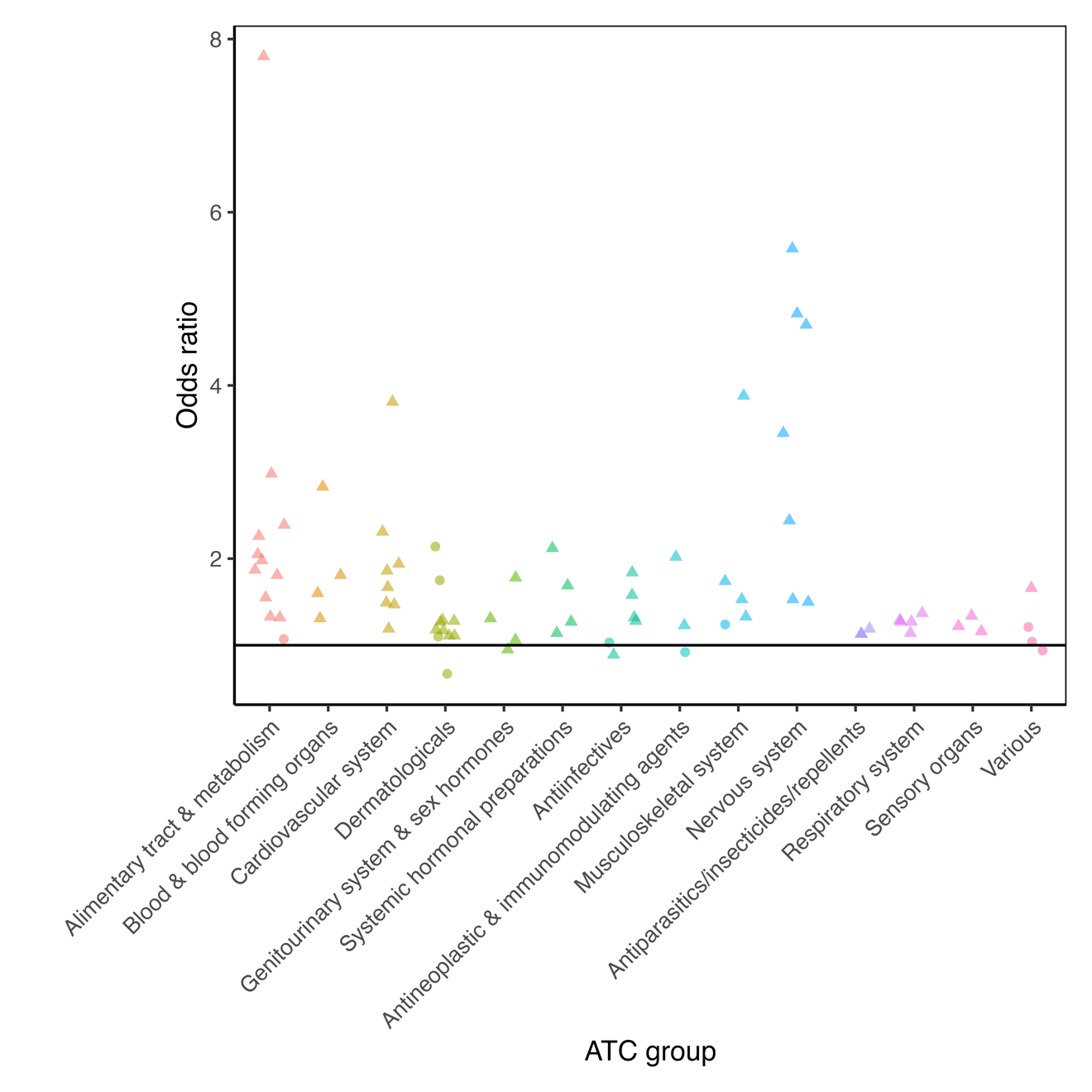
**

**Figure S11.** Association between other eating disorders and prescription medication in each of the Anatomical Therapeutic Chemical classification (ATC) chapters. *Note.* Significant results are indicated by a triangle whilst non-significant are circles.

**
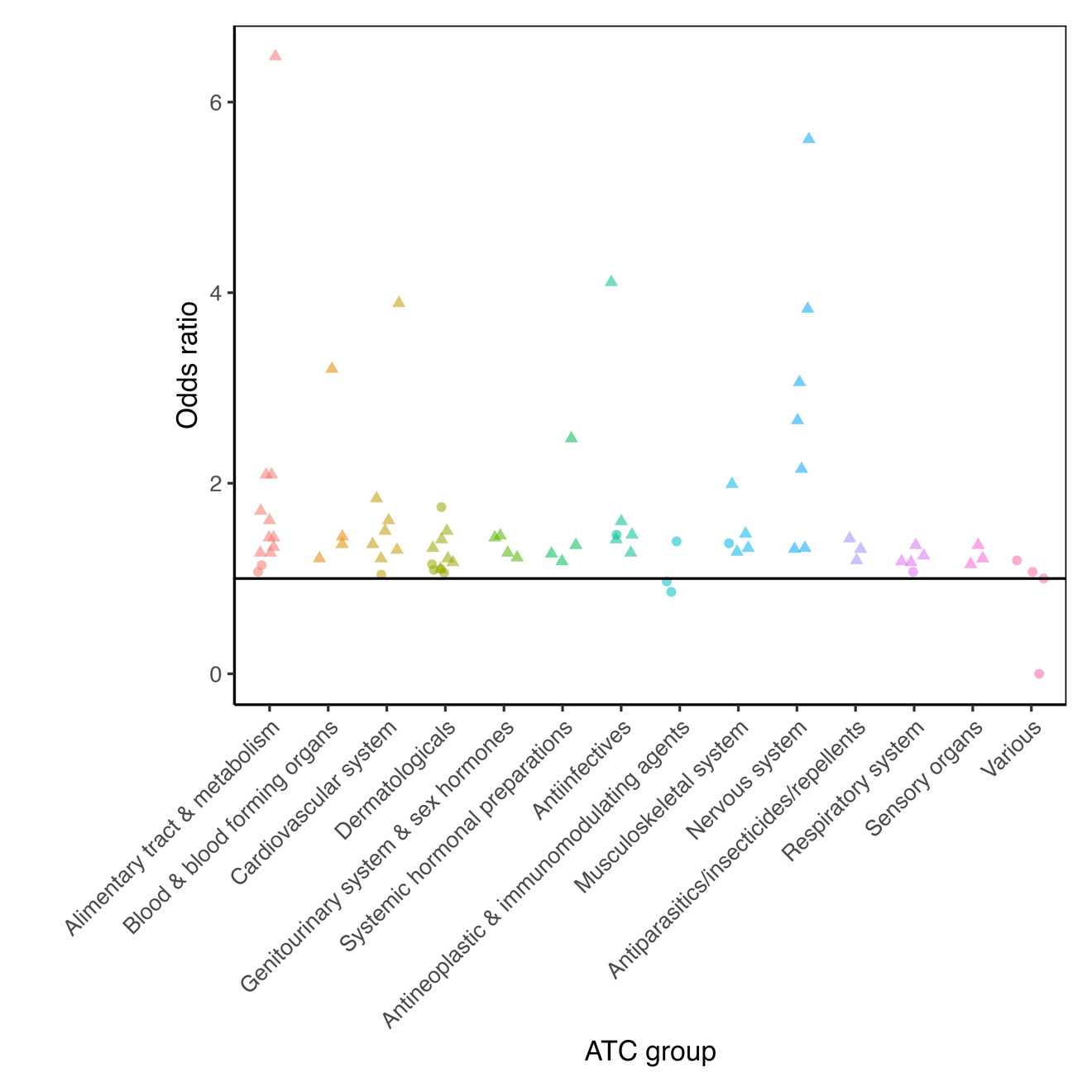
**

**Figure S12.** Association between bulimia nervosa and prescription medication in each of the Anatomical Therapeutic Chemical classification (ATC) chapters. *Note.* Significant results are indicated by a triangle whilst non-significant are circles.
